# Declining mortality from breast cancer in Yorkshire, 1983–1998: extent and causes

**DOI:** 10.1038/sj.bjc.6601614

**Published:** 2004-02-03

**Authors:** P Pisani, D Forman

**Affiliations:** 1 Northern & Yorkshire Cancer Registry and Information Service, Arthington House, Cookridge Hospital, Leeds, UK; 2 Unit of Descriptive Epidemiology, International Agency for Research on Cancer, 150 Cours Albert-Thomas, Lyons, France; 3 Unit of Epidemiology & Health Services Research, Medical School, University of Leeds, UK

**Keywords:** breast cancer, mammography, treatment, time trends, mortality, survival

## Abstract

Incidence and mortality data, stage of disease and treatment information for female breast cancer were obtained for the years 1975–1999 for the former Yorkshire Regional Health Authority area, from the Northern and Yorkshire Cancer Registry and Information Service. Deaths by age group and 3-year calendar period were separated into those occurring among cases diagnosed 0–2 and 3–4 years preceding death (short- and medium-term survivors, respectively), and among longer-term survivors and age-adjusted rates were calculated separately by survival time. The 3-year survival of cases incident in 1991–1999 and in 1982–1990 were compared, adjusting for stage and treatment. Breast cancer mortality in Yorkshire stopped increasing around 1983 and has since consistently declined, primarily among short- and medium-term survivors. The 3-year survival was significantly improved in cases diagnosed in 1991–1999, compared with 1982–1990, in all age groups (hazard ratio (HR)=0.75, 95% c.l. 0.71–0.78). The improvement was least in the 65+ years age group (HR=0.83, 95% c.l. 0.79–0.88), intermediate in the youngest (<50 years) cases (HR=0.71, 95% c.l. 0.63–0.80) and greatest in the age group 50–64 years, offered routine screening after 1988 (HR=0.51, 95% c.l. 0.47–0.57). The benefit for cases diagnosed in the 1990s persisted, but was reduced after adjustment for stage in cases <65 years, while it disappeared in older cases (HR=1.01). Below age 65 years, the use of systemic therapy increased substantially, but did not explain the residual improvements in short-term prognosis. A greater decline in breast cancer mortality in Yorkshire from 1982 to 1984 was observed among short- and medium-term, than in longer-term, survivors. Much of the improvement in survival in cases <65 years could be attributed to a more favourable stage at diagnosis, whereas this accounted for nearly all the improvement in survival among older cases. Systemic therapy had little or no impact on 3-year survival. Continuing declines in mortality can be expected in the current decade, as a result of the long-term effects of both mammography screening and increased use of systemic therapy.

In the mid-1980s, England and Wales had the highest age-standardised mortality rate for female breast cancer in the world (World Health Organisation, 1985, 1986, 1987), most likely the result of relatively high incidence rates and poor prognosis of disease at diagnosis. Decreases in breast cancer mortality rates during the 1990s have been observed in several countries (Botha *et al*, 2003), but the decline in the UK, that began in the mid-1980s, has been dramatic (Beral *et al*, 1995; Quinn and Allen, 1995; Hermon and Beral, 1996; Peto *et al*, 2000). This has largely been attributed to improved prognosis, as there has been no evidence of a decrease in the underlying risk of disease (Peto *et al*, 2000). The main determinants of prognosis are early stage at diagnosis and efficacy of treatment delivery. Assessment of the specific quantitative impact of these two determinants requires information on both the stage at diagnosis and of different treatment regimens among cases. We have conducted such an analysis for cases resident in the former Yorkshire Regional Health Authority area using data obtained from the Northern and Yorkshire Cancer Registry and Information Service (NYCRIS). This area covers a socially diverse, stable population of approximately 3.6 million.

## MATERIALS AND METHODS

### Analysis of mortality

The Office for National Statistics data on breast cancer deaths for residents in the former Yorkshire Regional Health Authority, by age and year, were obtained from NYCRIS. Mortality rates and their confidence intervals were age-standardised to the European standard population (Doll and Cook, 1967). A total of 20 489 deaths by age and 3-year calendar periods starting in 1976–1978 were separated into those occurring among cases diagnosed in the 3 years preceding death (short-term survivors, 0–2 years of survival), those among medium-term survivors (diagnosed 3–4 years before death) and those among longer-term survivors. As registration information was available only from 1975 onwards, deaths that occurred in 1975–1978 could not be categorised by survival time.

### Analysis of survival

Incidence data for primary female breast cancer (ICD10=C50) for the Yorkshire RHA population were extracted from the NYCRIS database for all completed years of registration from 1982 to 1999 (35 739 cases). We limited the survival analyses to cases diagnosed from 1982 onwards, in order to compare two periods of similar duration for women diagnosed during the 1980s, when mortality was still on the increase, and the 1990s when mortality declined. We excluded a further 507 cases recorded only on death certificate information or diagnosed at autopsy. Diagnoses of second primary cancers in the same patient (2711) were also excluded, leaving a total of 32 521 new diagnoses.

Tumour size, presence or absence of positive lymph nodes and of distant metastases were used to assign cases to one of the four standard stage groupings (Beahts *et al*, 1988). This was possible for 7997 (25%) of the cases. Partial TNM information was available for an additional 8912 (27%) cases, which were classified into two intermediate groups, one (termed I/II) including any tumours with size T1 and the other (termed II/III) including any tumours with size T2 or larger; both these groups excluded cases with evidence of metastases. Owing to the high correlation between tumour size and other parameters of stage (Duffy *et al*, 1991; Crisp *et al*, 1993), it was assumed that most cases in group I/II were either stage I or II and that group II/III included mainly stages II and III. The residual proportion of cases with no or insufficient information on stage was 48%.

Therapy administered within the first 6 months of diagnosis was recorded (yes or no) separately for surgery, radiotherapy, chemotherapy and hormonal therapy. Data were over 98% complete for these four indicator variables. Since information on stage was available for only 52% of cases, we could not evaluate the incidence trends by stage. The 3-year survival of cases incident in 1991–1999 was compared with that of cases incident in 1982–1990 using Cox regression. The extent of improvement in short-term survival due to advanced diagnosis was then evaluated by the effect of adjustment for stage. Improvements in survival due to treatment factors were also evaluated, adjusting by the indicator variables for each of the therapeutic modalities. Hazard ratios (HR) comparing the above two periods were calculated separately for the three age groups <50, 50–64 and 65+ years, adjusted for age (single year) within each interval.

## RESULTS

### Trends in mortality rates

**Figure 1:**
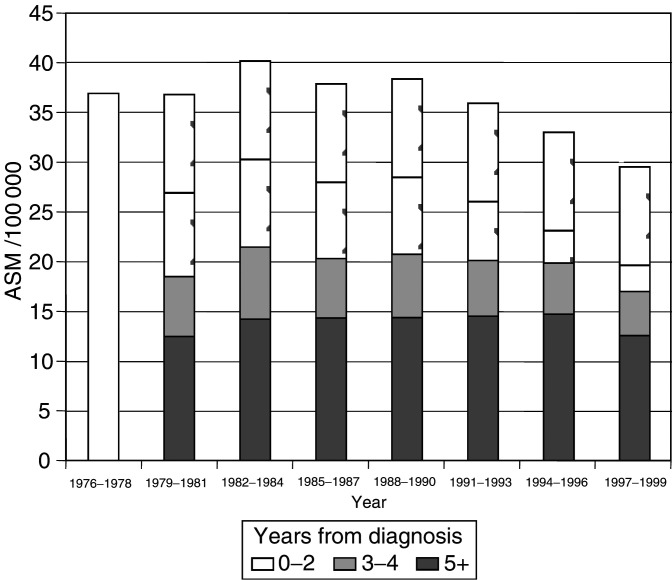
Age-standardised mortality rates for female breast cancer by time period and years from diagnosis, Yorkshire.

Age-standardised mortality rates (ASMRs) by calendar periods of 3 years and years since diagnosis are shown in Figure 1

. The decline of mortality began around 1985. Age-standardised mortality rates in 1982–1984 to 1997–1999, overall and by survival time, are given in Table 1

**Table 1:** Female breast cancer age-standardised mortality rates (ASMR) per 100 000 and 95% confidence intervals in 1982–1984 and 1997–1999 by years since diagnosis – Yorkshire (former Regional Health Authority Area)

	**ASMR**				
**Years since diagnosis**	**1982–1984**	**1997–1999**	**Change**	**% Change**	***P***
0–2	18.7	12.5	−6.2	−33.2	0.000
	(17.6-19.8)	(11.6-13.4)			
3–4	7.2	4.4	−2.8	−38.9	0.000
	(6.5-7.9)	(3.9-5.0)			
5+	14.3	12.6	−1.7	−11.9	0.008
	(13.3-15.2)	(11.7-13.4)			
All	40.1	29.5	−10.6	−26.5	0.000
	(38.5-41.8)	(28.2-30.8)			

. Overall, the rate decreased by 11 deaths/100 000 population, corresponding to 26% of the initial rate. This decrease was largely confined to short- and medium-term survivors (cases diagnosed within the 5 years preceding death), with statistically significant declines of around 35%. The decrease in longer-term survivors, although statistically significant, was much smaller in magnitude, around 12%. We observed the same pattern of mortality reductions over time within each of the three age groups <50, 50–64 and 65+ years (details not shown). Comparison of the two time periods considered in the survival analysis (see below) showed a reduction in the ASMR from 38.8/100 000 in 1982–1990 to 32.8/100 000 in 1991–1999, with statistically significant declines of around 35%.

### Trends in stage distribution

Figure 2

**Figure 2:**
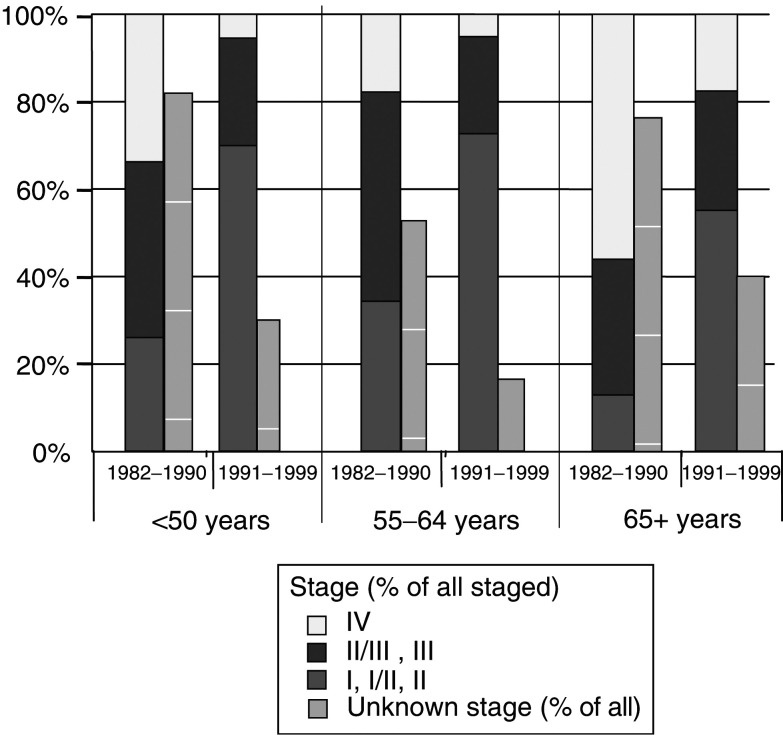
Relative distribution of stage at diagnosis and proportion of cases of undetermined stage for female breast cancer by time period and age group, Yorkshire.

shows the relative distribution of defined stages and of undetermined stage, by age and time period. The proportion of patients diagnosed with advanced stage disease (II/III–IV) decreased substantially in the 1990s at all ages, by 40% on average. The proportion of cases with no recorded information on stage was the lowest (33% on average in 1982–1999) in the 50–64 age group, particularly in the second period when this group was the target of mammography screening. The proportions with missing stage data were similar in the other two age groups, 53% in <50 years and 57% in 65+ years. The stage-specific 5-year survival rates, estimated by the Kaplan–Meier method, are shown in Table 2

**Table 2:** Number of female breast cancer cases and proportion surviving 5 years (Kaplan–Meier estimate) by stage at diagnosis^a^ – Yorkshire, 1982–1999

**Stage**	**No.**	**Proportion surviving 5 years**	**95% c.l.**
*Cases incident in 1982–1990*			
I	181	0.86	(0.80–0.90)
I/II	842	0.84	(0.82–0.87)
II	122	0.66	(0.57–0.74)
II/III	1761	0.63	(0.61–0.66)
III	69	0.39	(0.28–0.50)
IV	1395	0.12	(0.10–0.13)
All stages	4370	0.52	(0.50–0.53)
Insufficient or no information	10 460	0.60	(0.59–0.61)
			
*Cases incident in 1991–1999*			
I	685	0.91	(0.94–0.98)
I/II	3518	0.86	(0.85–0.88)
II	3434	0.73	(0.70–0.75)
II/III	2195	0.67	(0.65–0.69)
III	749	0.61	(0.56–0.66)
IV	1111	0.15	(0.13–0.18)
All stages	11 692	0.70	(0.69–0.71)
Insufficient or no information	5043	0.58	(0.57–0.60)

a See text for stage definitions.

for 1982–1990 and 1991–1999. The survival of patients in group I/II was intermediate between that of stages I and II, and survival in group II/III was intermediate between stages II and III in both time periods. For cases incident in the 1980s, the 5-year survival of staged cases was significantly lower than that of unstaged cases (0.52%, 95% c.l. 0.50–0.53 *vs* 0.60, 95% c.l. 0.59–0.61). *Vice versa*, survival of cases diagnosed in the 90s was superior among those staged (0.70%, 95% c.l. 0.69–0.71), compared to those unstaged (0.58%, 95% c.l. 0.57–0.60). Survival of cases in the second time period was improved for every stage except stage IV.

### 3-year survival comparison

Overall, patients diagnosed in the period 1982–1990 had a 3-year survival rate of 69%. This increased to 90% for patients diagnosed in 1991–1999. Table 3

**Table 3:** Proportional hazard ratios (Cox regression) for 3-years survival from female breast cancer for patients diagnosed in 1991–1999 compared to 1982–1990 – Yorkshire

		**Adjustments**		
**Age group**	**No. of cases**	**Age (single years)**	**Age & stage**	**Age, stage, hormone treatment, chemotherapy**
All^*^	32 521	0.75	0.95	0.95
		(0.71–0.78)	(0.90–0.99)	(0.91–1.00)
<50 years	7347	0.71	0.80	0.71
		(0.63–0.80)	(0.70–0.91)	(0.61–0.83)
50–64 years	10 947	0.51	0.68	0.65
		(0.47–0.57)	(0.61–0.76)	(0.59–0.74)
65+ years	14 227	0.83	1.01	1.06
		(0.79–0.88)	(0.96–1.08)	(1.00–1.13)

shows the hazard ratios (3-year survival) of cases diagnosed in 1991–1999 compared with cases diagnosed in 1982–1990. Overall, there was a statistically significant improvement in the later time period (HR=0.75, 95% CI 0.71–0.78), but was substantially reduced after adjustment for stage (HR=0.95, 95% CI 0.90–0.99).

When analysed by age group, the improvement in prognosis was more evident in women below 65 years of age, and was highest in the age group eligible for screening, 50–64 years (HR=0.51, 95% CI 0.47–0.57). The difference between the two time periods was lowest in the 65+ years age group, but remained statistically significant (HR=0.83, 95% CI 0.79–0.88). When stage was included in the model, the HR differences between the two time periods persisted below age 65 years, although they were reduced to 0.80 (95% CI 0.70–0.91) below 50 years and 0.68 (95% CI 0.61–0.76) at 50–64 years. In women 65+ years, stage at diagnosis accounted for all the improvement in survival, and the adjusted HR was 1.01 (95% CI 0.96–1.08).

### Changes in treatment patterns

Figure 3

**Figure 3:**
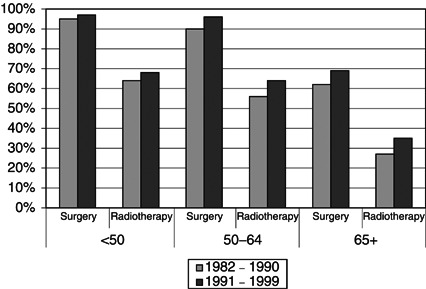
Percent of cases receiving surgery and radiotherapy for female breast cancer by time period and age group, Yorkshire.

shows for the three age groups, only a relatively small increase between 1982–1990 and 1991–1999 in the proportion of breast cancer patients undergoing surgery, 90–95% in women <65 years and 60–70% in women 65+ years. The small increase was very similar for the proportion of women receiving radiotherapy. In contrast, there were substantial increases in the use of systemic therapy between the two time periods (Figure 4

**Figure 4:**
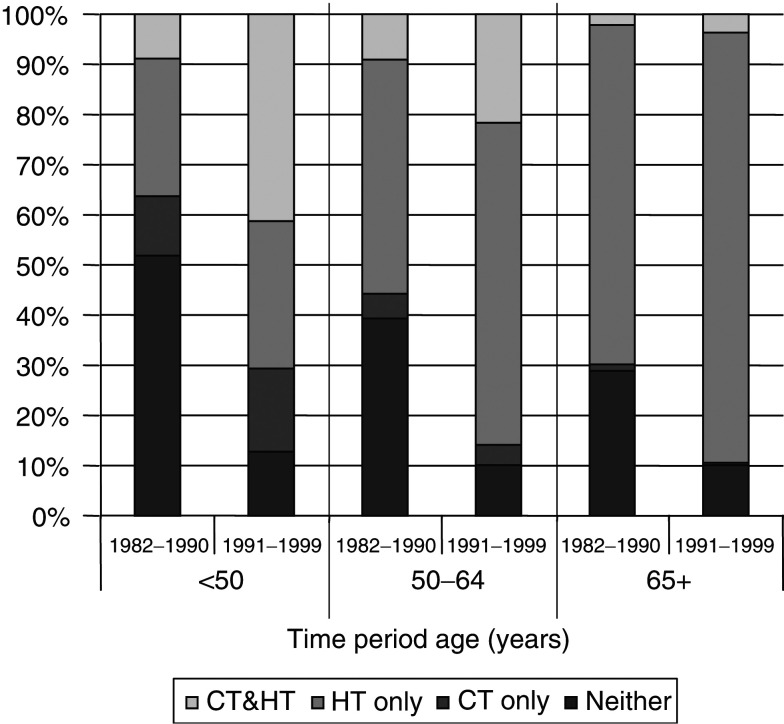
Proportions of cases receiving chemotherapy (CT) and/or hormone therapy (HT) for female breast cancer by time period and age group, Yorkshire.

). In the earlier period, approximately 50% of cases less than 50 years did not receive any systemic therapy, 10% had chemotherapy only and 40% had hormone therapy, that in 9% of the cases was combined with chemotherapy. By the 1990s, only 13% of cases in this age group had no systemic therapy, and the proportion treated with both hormone and chemotherapy increased to 41%. In all, 40% of cases aged 50–64 years in the 1980s received no systemic therapy, and hormone therapy was more common among those treated (56%). The proportion of cases that did not receive systemic therapy was reduced to 10% in the 1990s, almost entirely due to increased use of hormone therapy. In cases over 65 years, the use of chemotherapy was rare in either period (<5% of all cases) and hormone therapy, that was already common in the 1980s (70% of the cases treated), increased to 90% of all cases. In summary, the most important change observed over time was the increase in the proportion of cases receiving hormone therapy particularly under age 65 years. Despite these substantial changes over time, adjustment for chemotherapy and hormone treatment had no statistically significant effect in any age group or overall (Table 3).

## DISCUSSION

Recent time trends in the incidence and mortality of breast cancer in Yorkshire follow the general pattern of that reported for the UK overall (Northern & Yorkshire Cancer Networks, 1999; Blanks *et al*, 2000). Incidence and mortality trends have been diverging since the mid-1980s, indicating important improvements in the prognosis of the disease. The aim of this study was to evaluate the causes of the decline in mortality, taking advantage of the information on stage and treatment available in the NYCRIS database.

Stage-specific and treatment-specific trends of mortality rates would be the ideal measures to separate the impact of early diagnosis and treatment, but this would require complete information throughout the period studied. Owing to the incompleteness of data on stage, we used short-term survival as the outcome measure as recently proposed (Stockton *et al* 1997). In the presence of trends towards early diagnosis, survival time is artificially prolonged by lead time, producing spurious improvements of prognosis over time. Mortality *per se* is, in contrast, not affected by lead time, and thus the observed overall 26% decline is an unbiased finding. We have also shown that the decline in breast cancer mortality in Yorkshire from 1982 to 1984 appears to be largely confined to short- and medium-term survivors in each of the three age groups considered. Although these analyses are still biased by lead time (when diagnosis is advanced, more deaths are attributed to longer survival), an average lead time of several years would be required to account for all the observed effect. We believe, therefore, that the mortality decline is primarily related to prognostic improvements in the 5 years after diagnosis. This should be investigated further by investigation of the quantitative impact on mortality of shifts in stage and changes in treatment patterns, taking into account the results of randomised trials. Quantitative estimates using different scenarios would help a full interpretation of our results as well as the general trends. We plan to conduct further research on this subject using simulations.

In the 1980s, a large proportion of cases were diagnosed at advanced stages, and this proportion was reduced substantially in the subsequent decade (Figure 2). Overall, nearly all the difference in survival was accounted for by stage adjustment, and the benefit in terms of reduction of mortality was evident at all ages. Very similar results were reported for East Anglia (Stockton *et al*, 1997). In our data, a residual advantage for cases below age 65 years and diagnosed in the 1990s persisted and remained statistically significant after adjustment for clinical stage.

We also found quite different patterns of treatment in the two time periods, with substantial increases in the use of systemic therapy. These changes did not, however, explain the residual short-term improvements over time. This lack of effect on mortality is consistent with findings from a major overview of randomised trials of early breast cancer, in which the reduction in mortality at 3 years of follow-up for treated cases was less than 3% (Early Breast Cancer Trialists' Collaborative Group, 1998a, 1998b). One would not, therefore, expect to detect the effects of systemic therapy in 3 years of follow-up in a much more heterogeneous population of cases that includes advanced disease and not all benefiting from optimal treatment. Short-term effects of systemic therapy were not detected in other population-based data (Nab *et al*, 1994; Stockton *et al*, 1997). Our data also suggest a worse survival, albeit not statistically significant, associated with systemic therapy in the youngest age group. The most likely interpretation of this observation is that this is the result of a tendency to administer chemotherapy and endocrine therapy to cases of poorer prognosis within our broad stage categories.

Two factors may explain the residual difference in survival of younger cases between the two time periods. There was a large number of unstaged cases in our data set and, even among those staged, there may be inaccuracies. The proportion of unstaged cases decreased substantially over time, and it may be that the process of stage characterisation has similarly improved over time. This could, therefore, result in a differential stage misclassification bias such that, in the later time period, cases are more likely to be categorised as advanced cancers that, in the earlier time period, would be considered as ‘early’. This phenomenon, when determined by changes in clinical practice, has been termed as ‘stage migration’ (Feinstein *et al*, 1985). Improved accuracy of stage description over time would result in an apparent stage-specific improvement in survival. This phenomenon would be the greatest for cases in the screening age group. The standard classification of stage describes the progression of clinical disease, but does not make provision for the many subclinical tumours that are detected by mammography. If invasive, these are pooled with stage I clinically detectable tumours. In cases 50–64 years of age diagnosed in the 1990s, the stage I group includes many invasive screen-detected subclinical cancers, whose survival is prolonged by lead time. Indeed, it is among these cases that the largest difference persists.

The second factor could be a real initial, albeit small, effect of screening that was not addressed in our analysis. A change in the mortality trend began before the first round of mammography examinations was completed in 1994. Mammography itself cannot therefore be the cause of the initial reduced risk of death, but it may account for some of the decline from 1995 onwards. Direct evidence that the NHS programme reduced the incidence of advanced disease as well as the mortality rate in women 50–54 years when first screened has been recently reported (Threlfall *et al*, 2003). Clearly, this would not apply directly to the age group <50 years, who were not invited for mammography although improvements in breast cancer prognosis, across all age groups, are likely to result from the implementation of the national screening programme that facilitated generalised improvements in breast cancer treatment and care.

In summary, we attribute most of the decline of mortality in Yorkshire in the 1990s to a generalised shift towards more favourable stages at diagnosis and earlier treatment. This is not predominantly an effect of screening. We also describe important changes in treatment regimens in the two periods compared, which, however, had no impact on short-time survival. Continuing declines in mortality can be expected in the current decade as a result of the long-term effects of both mammography screening (IARC, 2002; Threlfall *et al*, 2003) and increased use of systemic therapy (Early Breast Cancer Trialists' Collaborative Group, 1998a, 1998b).
